# scafari: exploring scDNA-seq data

**DOI:** 10.1093/bioinformatics/btaf477

**Published:** 2025-09-02

**Authors:** Sophie-Marie Wind, Thea Reinkens, Yvonne Lisa Behrens, Sarah Sandmann

**Affiliations:** Institute of Medical Informatics, University of Muenster, 48149 Muenster, Germany; Department of Human Genetics, Hannover Medical School, 30625 Hannover, Germany; Department of Medical Genetics, University Hospital Oldenburg, 26133 Oldenburg, Germany; Institute of Medical Data Science, Otto-von-Guericke University Magdeburg, 39120 Magdeburg, Germany

## Abstract

**Summary:**

Recent advances in single-cell sequencing made it possible to not just analyze a cell’s individual expression pattern, but to gain insights into a single cell’s genome using the cutting-edge technology single-cell DNA sequencing. Mission Bio is, with the Tapestri platform, one of the few providers of this technology. So far, however, there is only little open-source software available for user-friendly processing and quality analysis of this data type. With scafari, we present a tool that offers easy-to-use data quality control as well as explorative variant analyses and visualization.

**Availability and implementation:**

scafari is implemented as an R Bioconductor package featuring a shiny application and is available at https://bioconductor.org/packages/scafari.

## 1 Introduction

The advent of single-cell sequencing represents a major breakthrough in molecular biology, enabling unprecedented resolution by dissecting tissue complexity down to individual cells. Considering DNA, single-cell DNA-sequencing (scDNA-seq) enables—in contrast to bulk experiments where the DNA of all cells in one sample is homogenized—to decipher the complex interplay of mutation profiles on single cell level. Thus, it is now possible to shed light on the previously inaccessible complexity of heterogeneous tissues like tumors. Even small cellular populations that cannot be detected by bulk sequencing may be identified, analyzed and clonal evolution of hematological neoplasms can be revealed (Evrony *et al.* 2021).

Recently, Mission Bio launched the scDNA-seq platform “Tapestri” (Mission Bio, South San Francisco, CA, USA), which was—among others—used to investigate the clonal evolution of juvenile myelomonocytic leukemia to secondary acute myeloid leukemia. The use of scDNA-seq allowed to identify residual tumor clones (Volchkov *et al.* 2023) and to study the mechanisms of tumor drug resistance (Morita *et al.* 2020, Peretz *et al.* 2021).

Raw sequencing data produced by the Tapestri platfrom can be pre-processed using Mission Bio’s Tapestri Pipeline (https://portal.missionbio.com/). Downstream analyses can be performed using Mission Bio’s analysis software Tapestri Insights, its Python library “mosaic” (https://github.com/MissionBio/mosaic), as well as the R packages “optima” (Pei *et al.* 2023) and “scDNA”(Miles *et al.* 2020). However, Tapestri Insights is a proprietary application, not available as open-source software. In contrast, mosaic is available as an open-source Python library. However, it does not offer an easy-to-use graphical user interface (GUI) but requires advanced computational expertise from its users. While the R packages offer a basic workflow for analysis, the functionalities they offer as well as options for visualization are highly limited. Moreover, neither package provides a GUI (see Table 1, available as supplementary data at *Bioinformatics* online).

To address this gap, we introduce scafari, an R Shiny application that streamlines the processing and analysis of scDNA-seq data provided in .h5 files, offering an intuitive GUI for interactive analyses.

## 2 Implementation

**Figure 1.**
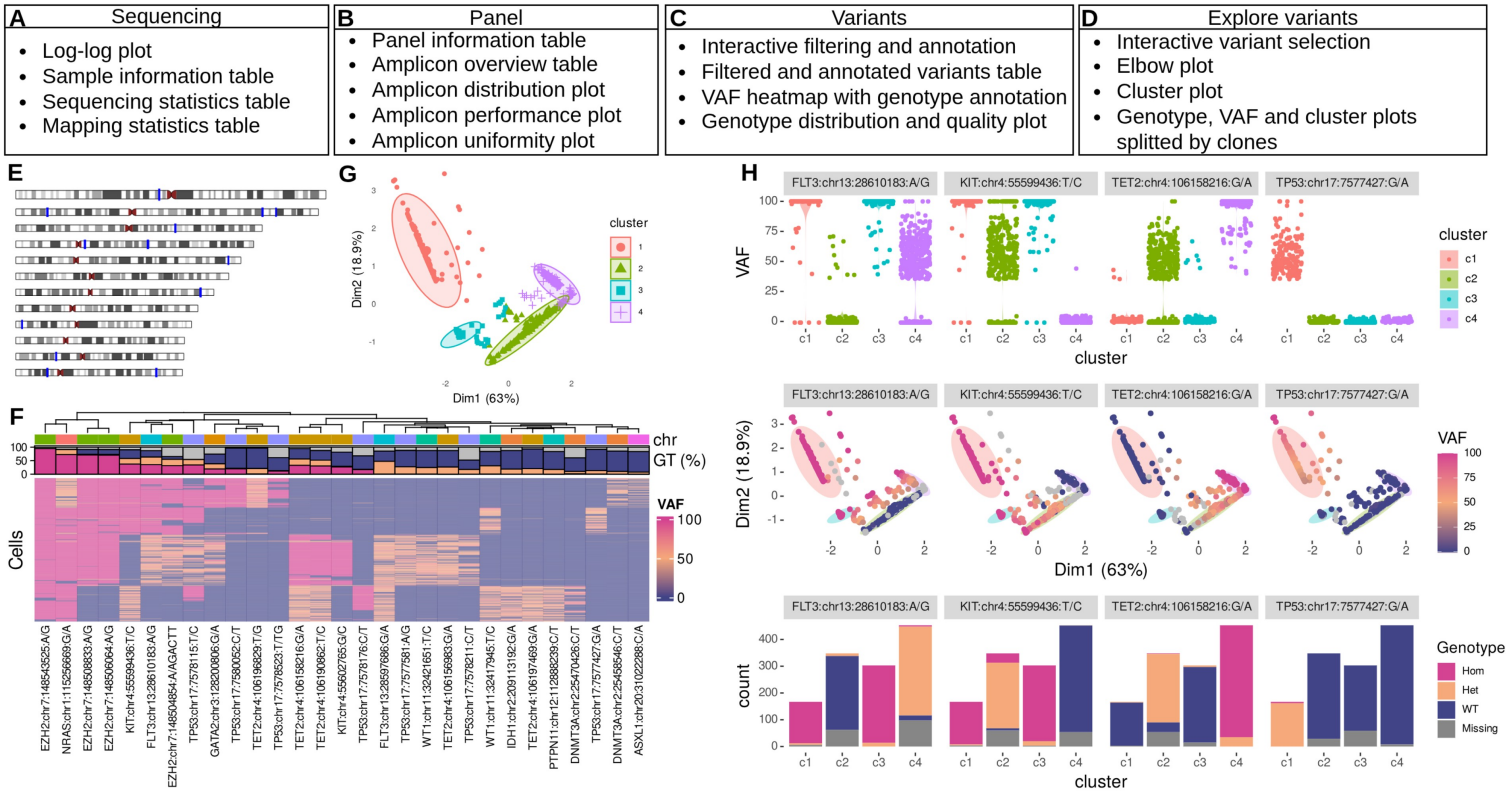
Schematic overview of scafari’s analysis workflow and exemplary results. The analysis is divided into four steps, represented by corresponding tabs: (A) “Sequencing,” (B) “Panel,” (C) “Variants” and (D) “Explore Variants.” An exemplary excerpt from a scafari analysis of test data shows (E) the amplicon distribution over the genome, (F) the VAF heatmap with the variants of interest over all filtered cells annotated with genotype, (G) the PCA of k-means clustered cells, and (H) the VAF distribution, PCA with noted VAFs per cell and genotype abundance for every selected variant of interest.

scafari is an R Bioconductor package for scDNA-seq analysis. The workflow is divided into four steps, represented by corresponding tabs: “Sequencing,” “Panel,” “Variants” and “Explore Variants.” A schematic overview of the analysis workflow is presented in Fig. 1.

In compliance with established data formats, scafari works on .h5 files, the standard output of the Tapestri pipeline. Subsequent to the file upload, a validity check is executed, ensuring that all relevant information is included in the file.

### 2.1 Sequencing

The “Sequencing” tab provides an overview of quality control metrics and important sequencing parameters. These include key information such as the total number of cells, the mapping rate, and the sequencing depth. The metrics allow the user to evaluate data quality, assess the overall performance of the experiment and identify potential issues that may have occurred (Fig. 1A; Figs 1 and 2, available as supplementary data at *Bioinformatics* online).

### 2.2 Panel

Information about the panel, amplicons and read counts, providing an outlook to the analysis of potential copy number variations, is included in the “Panel” tab (Fig. 1B). This includes, the panel name, as well as the number of amplicons and genes covered by the panel, among other things. The read counts are normalized, following the approach implemented in optima, and the amplicons annotated with information such as exon number and canonical transcript ID, supporting both GRCh37 [canonical transcripts from UCSC (Harrison *et al.* 2024)] and GRCh38 [Matched Annotation from NCBI and EMBL-EBI—MANE (Morales *et al.* 2022)].

Information about the annotated amplicons is provided in the “Amplicon overview” table (Fig. 3, available as supplementary data at *Bioinformatics* online). The exact genomic positions of the amplicons are represented in the “amplicon distribution plot” (Fig. 4, available as supplementary data at *Bioinformatics* online). Additional visualization options for assessing read counts per amplicon are available (Fig. 5, available as supplementary data at *Bioinformatics* online).

### 2.3 Variants

In the “Variants” tab, the variant calls present in the .h5 file can be analyzed to identify valid single nucleotide variants (SNVs; Fig. 1C). The first step of the analysis is the interactive variant filtering. Adapting the filtration approaches of mosaic and optima, filtering is performed on criteria such as sequencing depth, variant allele frequency (VAF) and genotype data. The thresholds can be adjusted by the user (Fig. 6, available as supplementary data at *Bioinformatics* online).

After filtering, the variants are annotated with biological information like gene names and clinical variant metrics. By default, annotation is performed via the Mission Bio API, which provides information on the names of the gene and the affected protein, the coding effect, the functional classification (e.g. intronic, coding), ClinVar information, the dbSNP ID and the DANN (Deleterious Annotation of genetic variants using Neural Networks) score. This score assesses the pathogenicity of genetic variants on a scale from 0 to 1, with higher values indicating a greater probability of deleteriousness (Quang *et al.* 2015).

If GRCh38 is used as reference genome, the Ensembl release 113 for GRCh38 (Harrison *et al.* 2024) is accessed with biomaRt and used for variant annotation (Durinck *et al.* 2009). Subsequent to filtering and annotation, the number of variants and cells that passed the filter as well as detailed annotation information is reported (Fig. 7, available as supplementary data at *Bioinformatics* online). To gain deeper insights into the VAF of filtered variants across the cells, a VAF heatmap with genotype annotations is available (Fig. 8, available as supplementary data at *Bioinformatics* online).

### 2.4 Explore variants

To further focus on selected variants of interest, the user can proceed with the analysis in the “Explore Variants” tab. Thereby, variants forming cell clusters/clones can be identified and analyzed regarding VAF and genotype (Fig. 1D).

Cells are clustered based on VAF. By default, k-means clustering is used, yielding superior results at the exemplary cell labeled dataset “50:50 Mix for CNV and SNV Analysis,” compared to alternative methods Leiden and DBSCAN (Fig. 9, available as supplementary data at *Bioinformatics* online). However, dependent on the number of variants selected for downstream analyses, alternative approaches may also lead to comparable results.

Depending on the clustering method selected, different visualizations are generated to assist in selecting the correct clustering parameters. Taking into account both the plots and experimental information, the user can define the clustering parameters (Figs 10 and 11, available as supplementary data at *Bioinformatics* online). Once clustering is completed, the variants of interest can be analyzed in terms of VAF and genotype across clusters using various visualization techniques, providing deeper insights into the genetic heterogeneity of the cells. These include a cluster plot (principal component analysis—PCA) and a clustered VAF heatmap (Fig. 12, available as supplementary data at *Bioinformatics* online).

To further evaluate the accuracy of clustering and to identify potential subpopulations, scatter plots with violin plots in the background are provided, showing the VAF distribution of filtered cells split by clusters. Additionally, cluster maps that are color-coded by VAF are included. These allow to visualize potential transitions and gradients between the clusters that may give insights in the population structure of the dataset. Finally, barplots show the genotype distribution per variant and per cluster (Fig. 13, available as supplementary data at *Bioinformatics* online).

## 3 Usage example

To demonstrate the functionalities of scafari, we analyzed the .h5 file of the Mission Bio’s “4 Cell Line Mix for CNV and SNV Analysis” dataset. The analyzed sample contained 1313 cells. An average of 32 read pairs per amplicon and cell was found (information available in the “Sequencing” tab; Fig. 1, available as supplementary data at *Bioinformatics* online).

Read count analysis, presented in the “Panel” tab, was conducted using Mission Bio’s “AML” panel, which includes 127 amplicons covering 20 genes (Fig. 3, available as supplementary data at *Bioinformatics* online). Investigating the distribution of the amplicons over the genome, targeted hotspot genes can be observed, e.g. *NRAS* on chromosome 1 (Fig. 1E; Fig. 4, available as supplementary data at *Bioinformatics* online). Performance evaluation shows high quality of the sample (Fig. 5, available as supplementary data at *Bioinformatics* online).

Investigating potential SNVs (“Variants” tab), 27 719 variants in 1313 cells were initially identified. Using scafari’s default filtering thresholds, 29 variants and 1271 cells remained (Fig. 6, available as supplementary data at *Bioinformatics* online). Filtered variants, including detailed annotation, are presented in the “Overview Filtered Variants” table (Fig. 7, available as supplementary data at *Bioinformatics* online).

Compared to default filtration with alternative approaches optima and mosaic, a high level of consistency can be observed (Tab. S2). While scafari and optima show perfect overlap, mosaic reports 11 additional variants, none of which are included in ClinVar or gnomAD. As 7 out of 11 equally lack a dbSNP ID, these might be artifacts.

In the gene *ASXL1*, as an example, the nonsense variant p. Y591* can be found. A DANN score of 0.9967 (highly deleterious) is assigned. These results are in line with the variant’s ClinVar annotation “Likely Pathogenic” and its low presence in gnomAD (7.9607 × 10^−6^).

Analysis of the VAF and genotype by heatmap shows that the non-sense variant in *ASXL1* (chr20:31022288C>A) has a VAF of 0% in a majority of cells (68%). Thus, these cells are characterized by wildtype *ASXL1*. However, 8% of the cells show a VAF between 35% and 95%. These were assigned with the genotype “heterozygous” (Fig. 1F; Fig. 9, available as supplementary data at *Bioinformatics* online).

Focusing on the four variants “KIT: chr4:55599436: T/C,” “TET2: chr4:106158216: G/A,” “FLT3: chr13:28610183: A/G,” and “TP53: chr17:7577427: G/A,” further detailed analyses were performed (“Explore variants” tab) (Fig. 10, available as supplementary data at *Bioinformatics* online). The elbow plot suggests, together with information about the dataset, an optimum k-means clustering with k=4 (Fig. 11, available as supplementary data at *Bioinformatics* online). The resulting plot shows successful clustering of the cells into four populations consistent with the four cell lines present in the dataset (Fig. 1G). A clustered VAF heatmap provides deeper insights. In accordance with the cluster plot, we observe distinct VAF patterns in the four groups (Fig. 12, available as supplementary data at *Bioinformatics* online).

A systematic comparison of the clusters c1, c2, c3 and c4 is performed in the “compare cluster” section of scafari. Considering the SNV in gene *FLT3* as an example, VAF of 100% can be observed in most cells in clusters c1 and c3. In cluster c2, however, VAF was ∼0% while in cluster c4 it ranged between 35 and 90%. This observation aligns with the variant’s VAF per cell visible in the PCA plot. No evidence for further subpopulations can be found. Derived genotype bar plots show a homozygous genotype for the *FLT3* variant being predominant in c1 and c3, wild-type in c2 and heterozygous in c4 (Fig. 1H; Fig. 13, available as supplementary data at *Bioinformatics* online).

## 4 Discussion and conclusion

We developed scafari, a Bioconductor package for user-friendly analysis of scDNA-seq data available as pre-processed .h5 files. At the example of data from Mission Bio’s Tapestri platform, we demonstrate scafari’s functionalities, supporting quality control, read count normalization, amplicon annotation, as well as variant filtering and characterization. Different from alternative approaches, mosaic, optima and scDNA, scafari provides the user with an extensive set of visualization techniques for exploring the results and to get deeper insights into the cellular heterogenity of scDNA-seq data, at runtime comparable to the fastest tool (mosaic). Furthermore, scafari is the only tool offering an intuitive GUI for data analysis, enhancing accessibility for users with limited computational expertise.

Currently, CNV calling is not yet supported by scafari. However, analysis of data quality and read-count distribution per amplicon represent an important pre-requisite. Similarly, by exploring variant calls and possible clustering, scafari provides the basis for subsequent lineage tree reconstruction. However, as complex branching tree structures are a widespread phenomenon in clonal evolution [e.g. 45% in AML (Morita *et al.* 2020); extensive branching structures in high-risk pediatric cancers (Andersson *et al.* 2020)], we consider their reconstruction a challenging analysis. Therefore, it is not implemented in scafari. However, we hope to address it as future work.

The field of single-cell omics is rapidly evolving, with scRNA-seq, spatial transcriptomics and scATAC-seq representing an option of analyzing multi-omics data on a single-cell level in near future. However, several challenges still remain, e.g. deconvolution of spatial transcriptomics data or cell-type annotation. Optimum integration of single-cell multi-omics data will be another focus of our future work.

## Supplementary Material

btaf477_Supplementary_Data

## Data Availability

The “4 Cell Line Mix for CNV and SNV Analysis” and cell labeled “50:50 Mix for CNV and SNV Analysis” are available at Mission Bios website: https://portal.missionbio.com/datasets/4-cell-lines-AML-CNV, https://portal.missionbio.com/datasets/2-cell-line-mix.

## Code availability

scafari is available at: https://bioconductor.org/packages/scafari.
