# Supplementary material for: scafari: exploring scDNA-seq data
Source: Bioinformatics. 2025 Sep 2;41(9):btaf477. doi: 10.1093/bioinformatics/btaf477 (PMC12449247; doi:10.1093/bioinformatics/btaf477)
Supplement: btaf477_Supplementary_Data [file btaf477_supplementary_data.pdf]

Supplementary Material for ‘scafari: Exploring scDNA-seq data’

1 Supplementary Figures

1.1 Sequencing tab

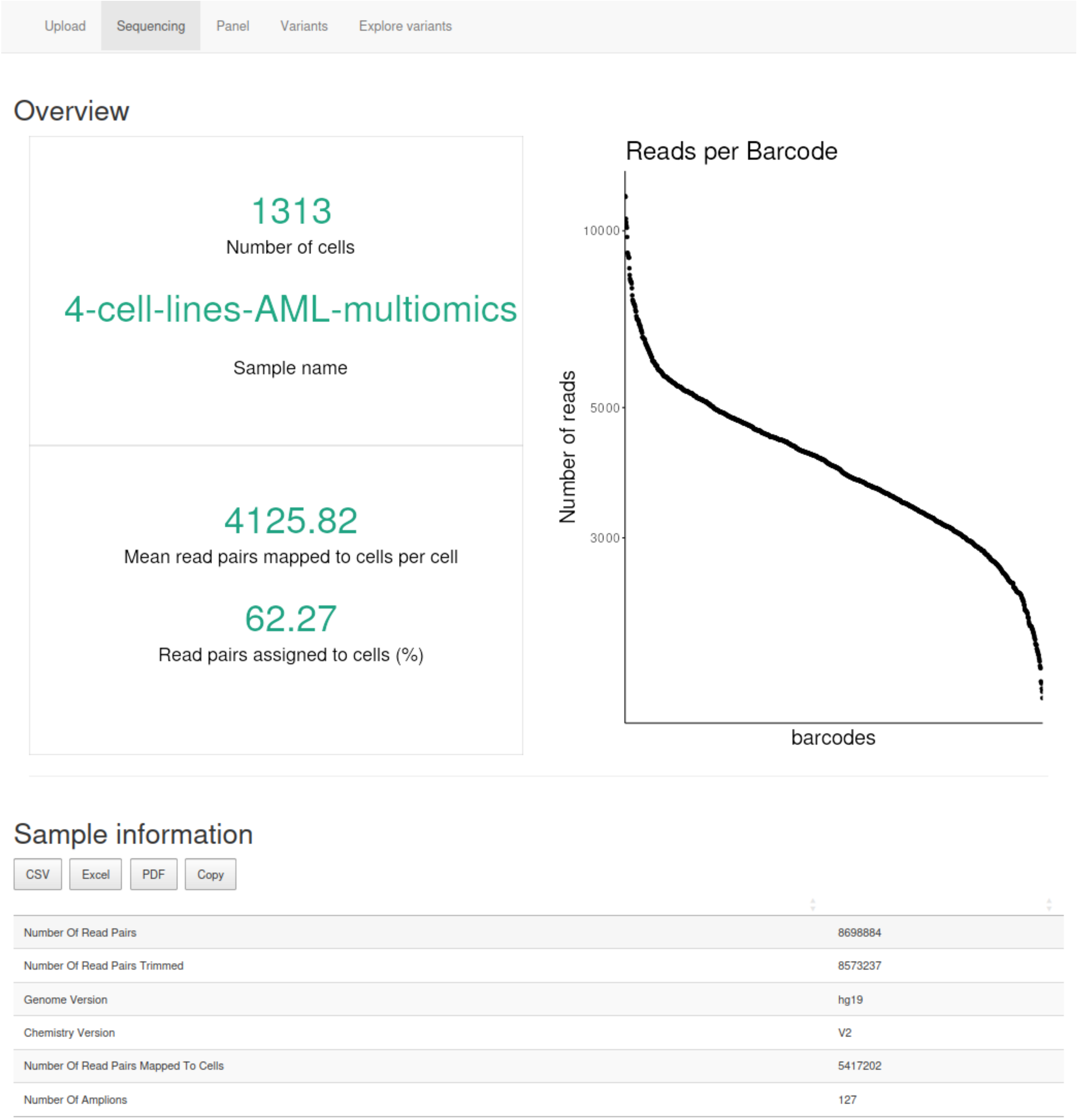

Figure S1: Summary of sequencing information. In scafari's "Sequencing" tab basic characteristics on data quality are provided, including a plot showing reads per barcode as well as detailed sample information.

Sequencing information

CSV Excel PDF Copy

Total read pairs	8698884
Read pairs trimmed	8573237
Read pairs with valid barcodes	8419726

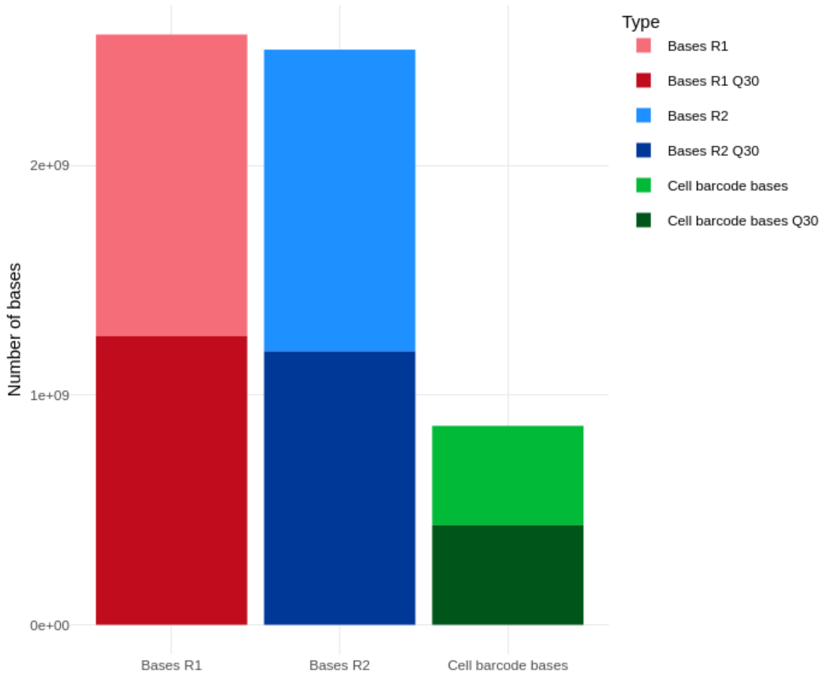

Mapping

CSV Excel PDF Copy

Reads mapped to genome (%)	94.57
Reads mapped to target (%)	79.19
Average mapping error rate (%)	0.68

Tapestri pipeline information

CSV Excel PDF Copy

Depth cutoff	10
Missing cells cutoff	50
Missing variants cutoff	50
Mutated cells cutoff	1

Figure S2: Summary of sequencing information (continued). In scafari's "Sequencing" tab additional information on sequencing and mapping, including a plot showing R1 vs R2 read distribution, is provided along with the Tapestri pipeline configuration.

1.2 Panel tab

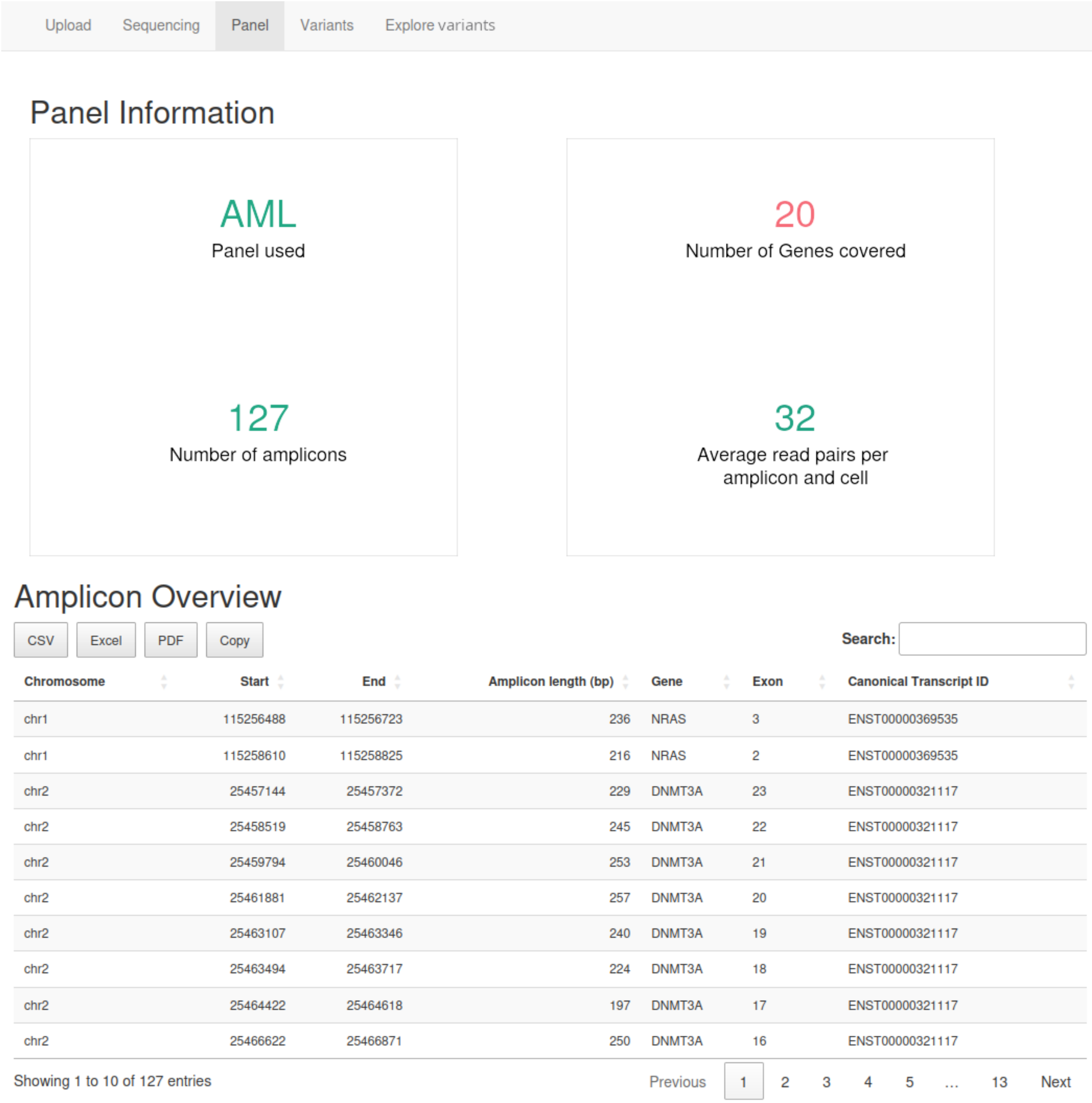

Chromosome	Start	End	Amplicon length (bp)	Gene	Exon	Canonical Transcript ID
chr1	115256488	115256723	236	NRAS	3	ENST00000369535
chr1	115258610	115258825	216	NRAS	2	ENST00000369535
chr2	25457144	25457372	229	DNMT3A	23	ENST00000321117
chr2	25458519	25458763	245	DNMT3A	22	ENST00000321117
chr2	25459794	25460046	253	DNMT3A	21	ENST00000321117
chr2	25461881	25462137	257	DNMT3A	20	ENST00000321117
chr2	25463107	25463346	240	DNMT3A	19	ENST00000321117
chr2	25463494	25463717	224	DNMT3A	18	ENST00000321117
chr2	25464422	25464618	197	DNMT3A	17	ENST00000321117
chr2	25466622	25466871	250	DNMT3A	16	ENST00000321117

Figure S3: Overview of sequencing panel and amplicons. Key information about the panel is summarized. Additionally, an interactive table provides more detailed information on the annotated amplicons, including the targeted gene, exon and canonical transcript ID.

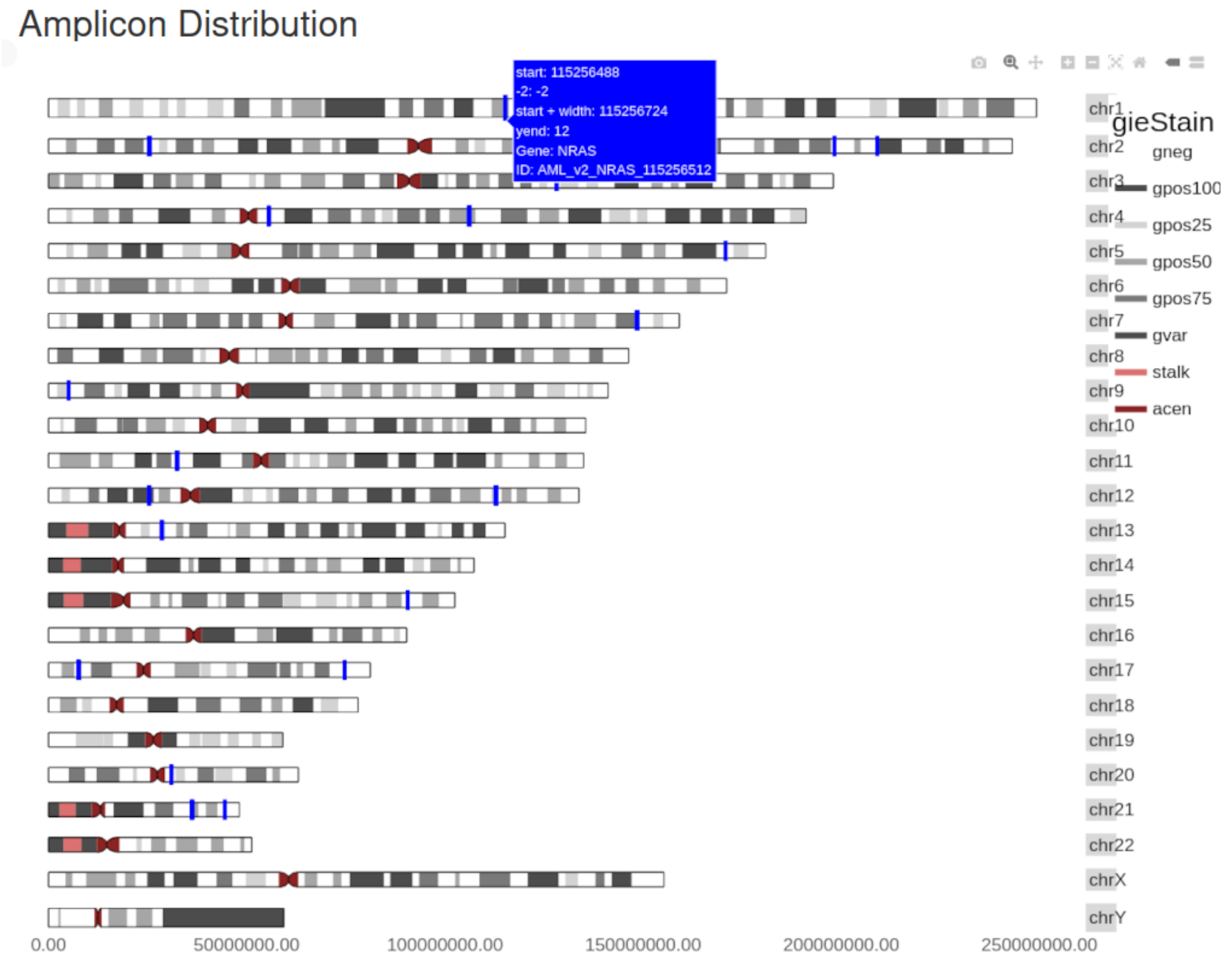

Figure S4: Amplicon distribution over the genome. The full human genome is displayed. Genes targeted by the panel are marked in red and labeled with the corresponding gene name.

Amplicon Performance

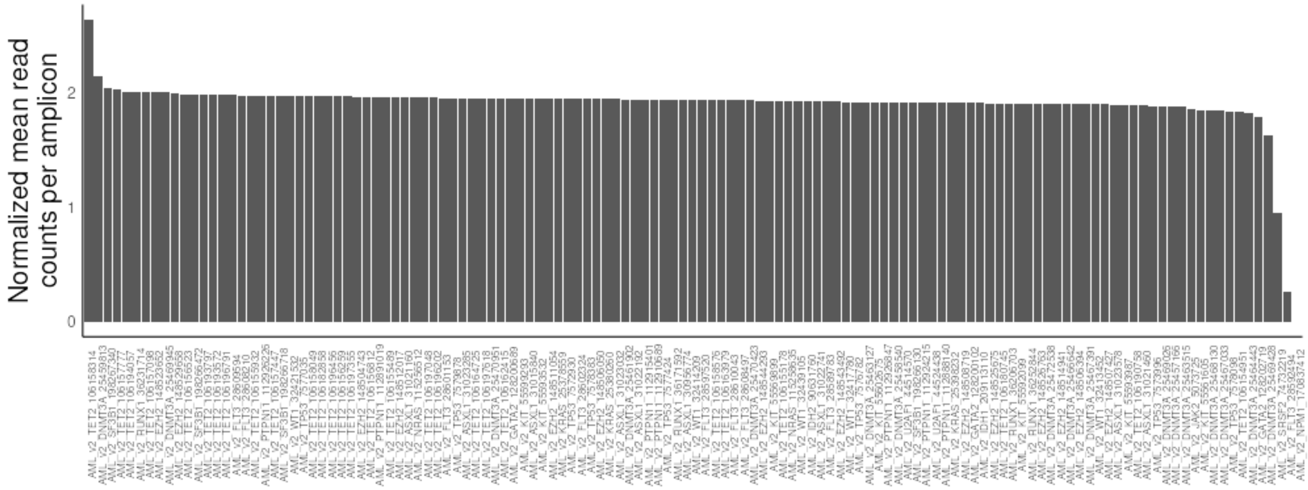

Panel uniformity plot

Panel Uniformity (%): 96.06

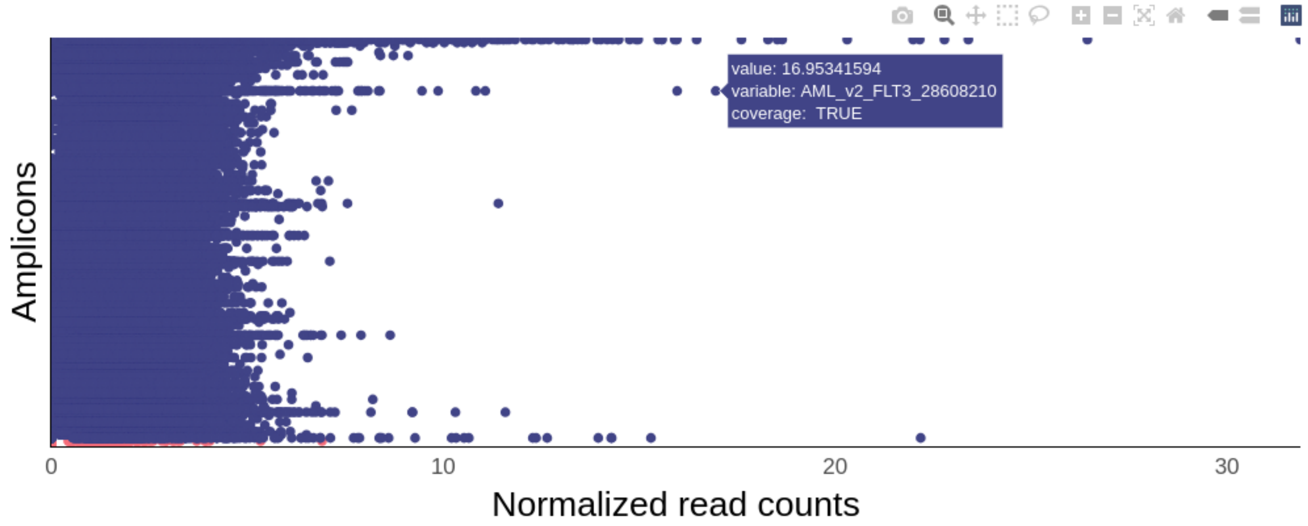

Figure S5: Amplicon performance and panel uniformity. Normalized read counts per amplicon are depicted in a bar plot. A panel uniformity plot is showing the normalized read counts per amplicon. Each row represents one amplicon and each dot one cell. The amplicons are sorted by their mean reads, from high to low. Amplicons meeting at least 20% of the average depth of coverage are represented in blue, others in orange.

1.3 Variants tab

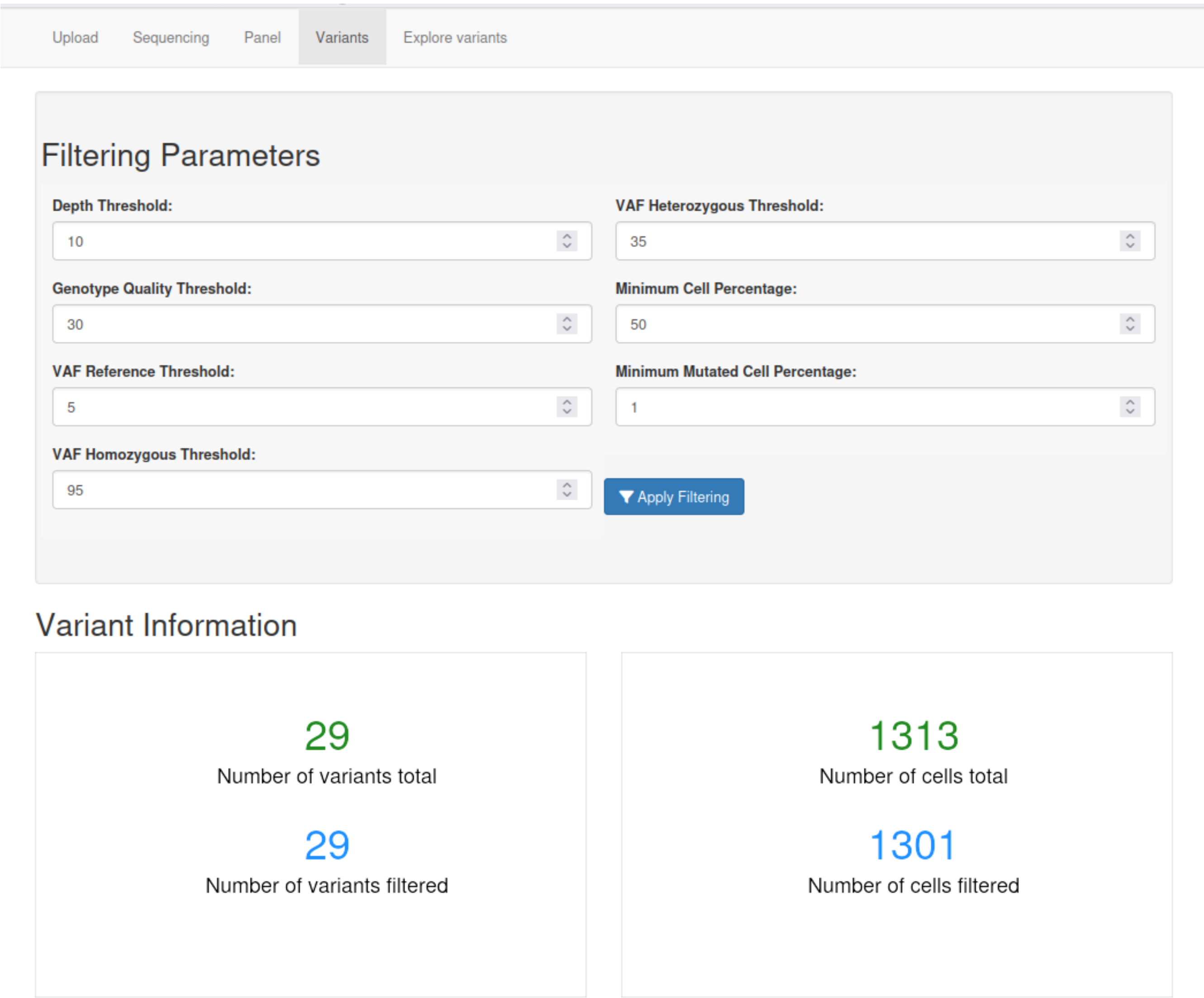

Figure S6: Interactive variant filtering. scafari provides an interactive interface with adjustable parameters for variant filtering (default settings displayed). Variant information, that is the number of cells and variants before and after filtering, are displayed below as the button 'Apply Filtering' is pushed.

Overview Filtered Variants

CSV Excel PDF Copy Search:

	Variant	Gene	Chromosome	Position	Alt	Ref	Protein	Coding.impact	Function	DANN	ClinVar	dbsnp	Allele Freq (gnomAD)
1	chr20:31022288:C/A	ASXL1	chr20	31022288	C	A	ASXL1:p.Y591*	nonsense	coding	0.996704000976592	Likely Pathogenic	rs371369583	7.96070595540412e-06
2	chr2:25458546:C/T	DNMT3A	chr2	25458546	C	T	DNMT3A		intronic	0.551042207098605	Benign	rs2304429	0.5137756208872
3	chr2:25470426:C/T	DNMT3A	chr2	25470426	C	T	DNMT3A		intronic	0.635212256233406	-	rs142243425	0.000702189852540131
4	chr7:148504854:A/AGACTT	EZH2	chr7	148504854	A	AGACTT	EZH2		intronic		Benign	rs560966145	
5	chr7:148506064:A/G	EZH2	chr7	148506064	A	G	EZH2		intronic	0.701239051484726	Benign	rs740949	
6	chr7:148508833:A/G	EZH2	chr7	148508833	A	G	EZH2		intronic	0.585497604297006	Benign	rs2072407	0.668850300386799
7	chr7:148543525:A/G	EZH2	chr7	148543525	A	G	EZH2		intronic	0.646504104739525	Benign	rs10274535	0.702331387055038
8	chr13:28597686:G/A	FLT3	chr13	28597686	G	A	FLT3		intronic	0.559099093600269	-	rs76991010	
9	chr13:28610183:A/G	FLT3	chr13	28610183	A	G	FLT3		-	0.544724875637074	Benign	rs2491231	0.709744290267692
10	chr3:128200806:G/A	GATA2	chr3	128200806	G	A	GATA2		intronic	0.915158543656728	Benign	rs11708606	0.156403002496676
11	chr2:209113192:G/A	IDH1	chr2	209113192	G	A	IDH1:p.G105=	synonymous	coding	0.680928983428449	Benign	rs11554137	0.0505702877686397
12	chr4:55599436:T/C	KIT	chr4	55599436	T	C	KIT		intronic	0.562532425916318	Benign	rs1008658	
13	chr4:55602765:G/C	KIT	chr4	55602765	G	C	KIT:p.L862=	synonymous	coding	0.469237342448195	-	rs3733542	0.10780608510232
14	chr1:115256669:G/A	NRAS	chr1	115256669	G	A	NRAS		intronic	0.485702078310495	Benign	rs969273	
15	chr12:112888239:C/T	PTPN11	chr12	112888239	C	T	PTPN11:p.H85=	synonymous	coding	0.542558061464278	Benign	rs61736914	0.00575481692115303
16	chr4:106156983:G/A	TET2	chr4	106156983	G	A	TET2:p.E628=	synonymous	coding	0.320337534714805	Benign	rs35695427	0.00414060505488895
17	chr4:106158216:G/A	TET2	chr4	106158216	G	A	TET2:p.S1039=	synonymous	coding	0.587786492507706	Benign	rs3796927	0.0321049314259728
18	chr4:106190862:T/C	TET2	chr4	106190862	T	C	TET2:p.H1380=	synonymous	coding	0.404873805963317	Benign	rs3733609	0.0341135840368381
19	chr4:106196829:T/G	TET2	chr4	106196829	T	G	TET2:p.L1721W	missense	coding	0.9315317239906	Benign	rs34402524	0.115576708011387
20	chr4:106197469:G/A	TET2	chr4	106197469	G	A	TET2:p.K1934=	synonymous	coding	0.414883877071444	Likely Benign	rs569967455	2.55261579303391e-05
21	chr17:7577427:G/A	TP53	chr17	7577427	G	A	TP53		intronic	0.690267647328105	Benign	rs12947788	
22	chr17:7577581:A/G	TP53	chr17	7577581	A	G	TP53:p.Y234H	missense	coding	0.998306222724082	-	rs864622237	
23	chr17:7578115:T/C	TP53	chr17	7578115	T	C	TP53		intronic	0.648030030213324	Benign	rs1625895	0.863335599365511
24	chr17:7578176:C/T	TP53	chr17	7578176	C	T	TP53		-	0.994476149784845	-	rs863224499	
25	chr17:7578211:C/T	TP53	chr17	7578211	C	T	TP53:p.R213Q	missense	coding	0.999435407574694	Pathogenic	rs587778720	3.97674400108167e-06

Showing 1 to 25 of 29 entries

Previous 1 2 Next

Figure S7: Overview of the filtered variants. Detailed information about filtered and annotated variants is additionally available in the "Variants" tab, including the coding impact, DANN score, ClinVar classification and dbSNP ID. Information is available for both hg19 (Mission Bio API) and hg38 (bimoaRt).

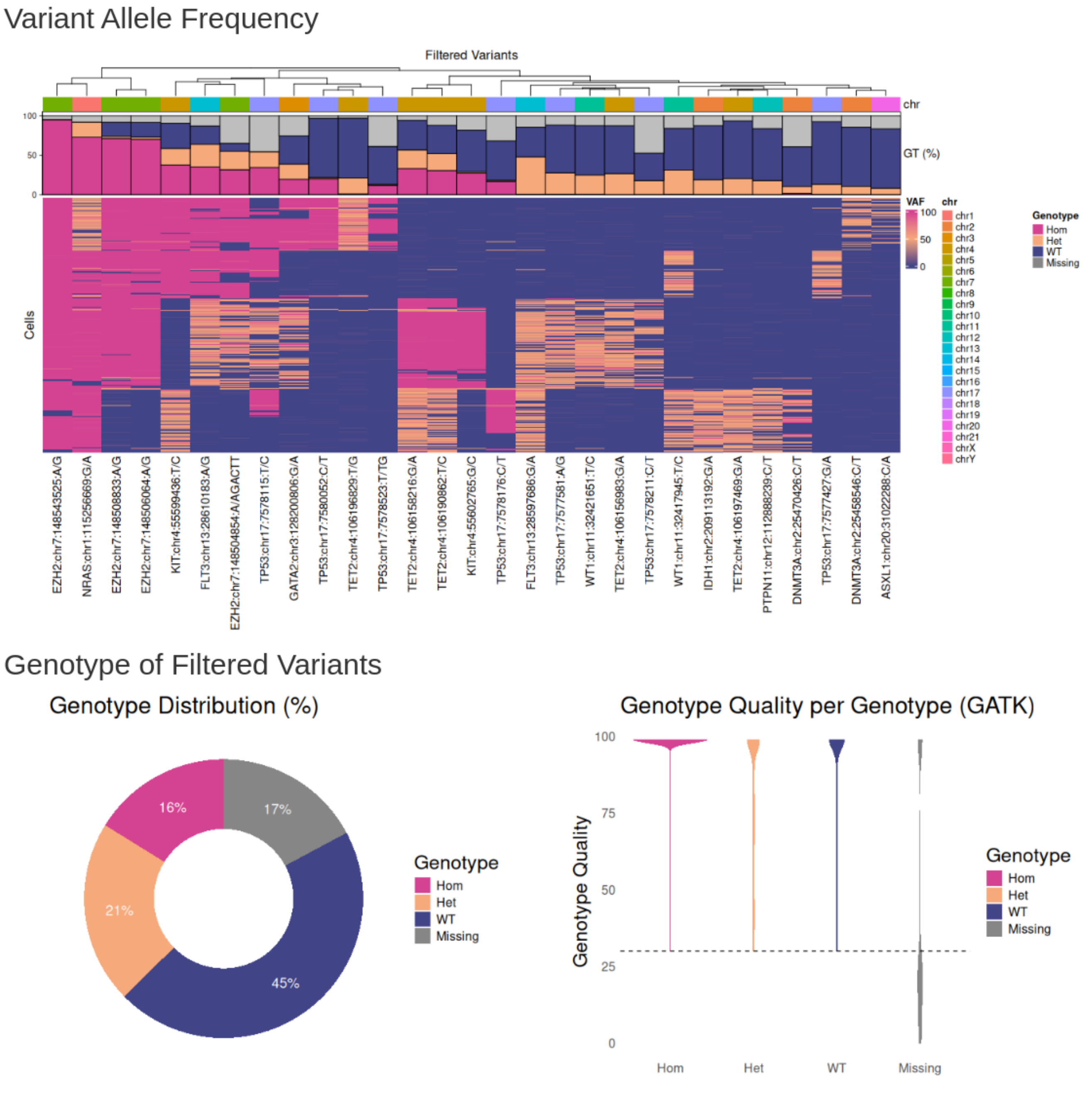

Figure S8: Variant allele frequency, genotype distribution and genotype quality of filtered variants. The variant allele frequency of the filtered variants is shown in a heatmap annotated with chromosome and genotype. A pie chart depicts the genotype distribution over filtered cells. Additionally, the genotype quality over all filtered cells can be evaluated in a violin plot.

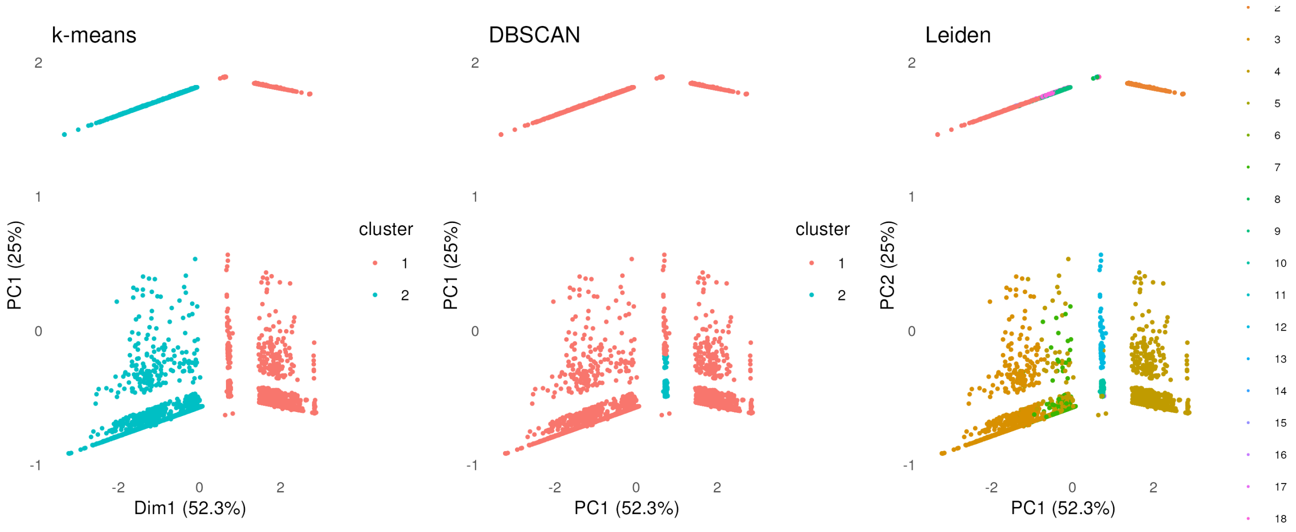

Figure S9: Variant clustering comparison using labeled "*50:50 Mix for CNV and SNV Analysis*". Cells are clustered by VAF using k-means, DBSCAN and Leiden clustering.

1.4 Explore profiles tab

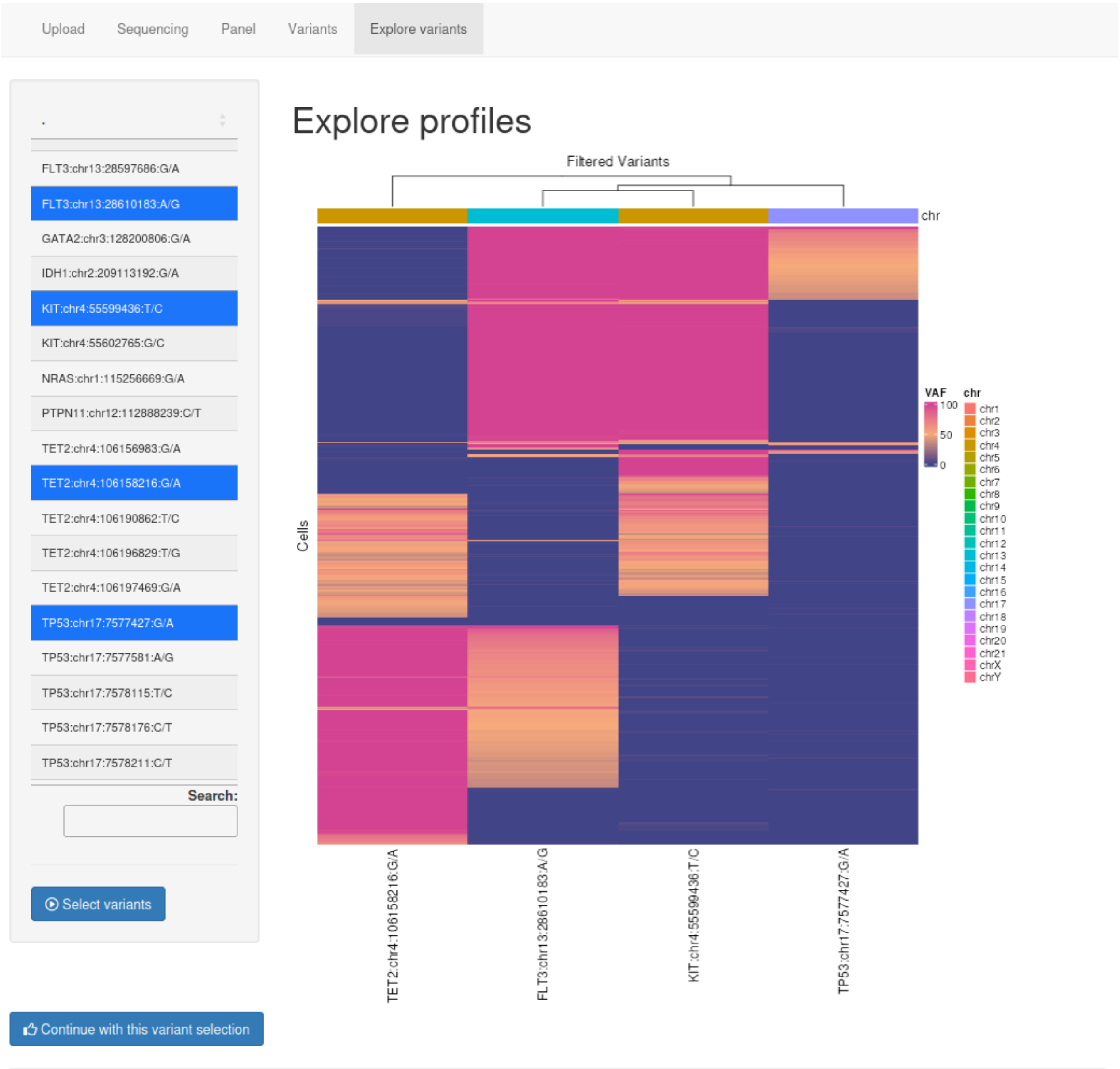

Figure S10: Interactive selection of the variants of interest. An individual selection of the variants of interest can be made by the user (interactive table on the left). The variant allele frequency of the selected variants over all cells is depicted in a heatmap.

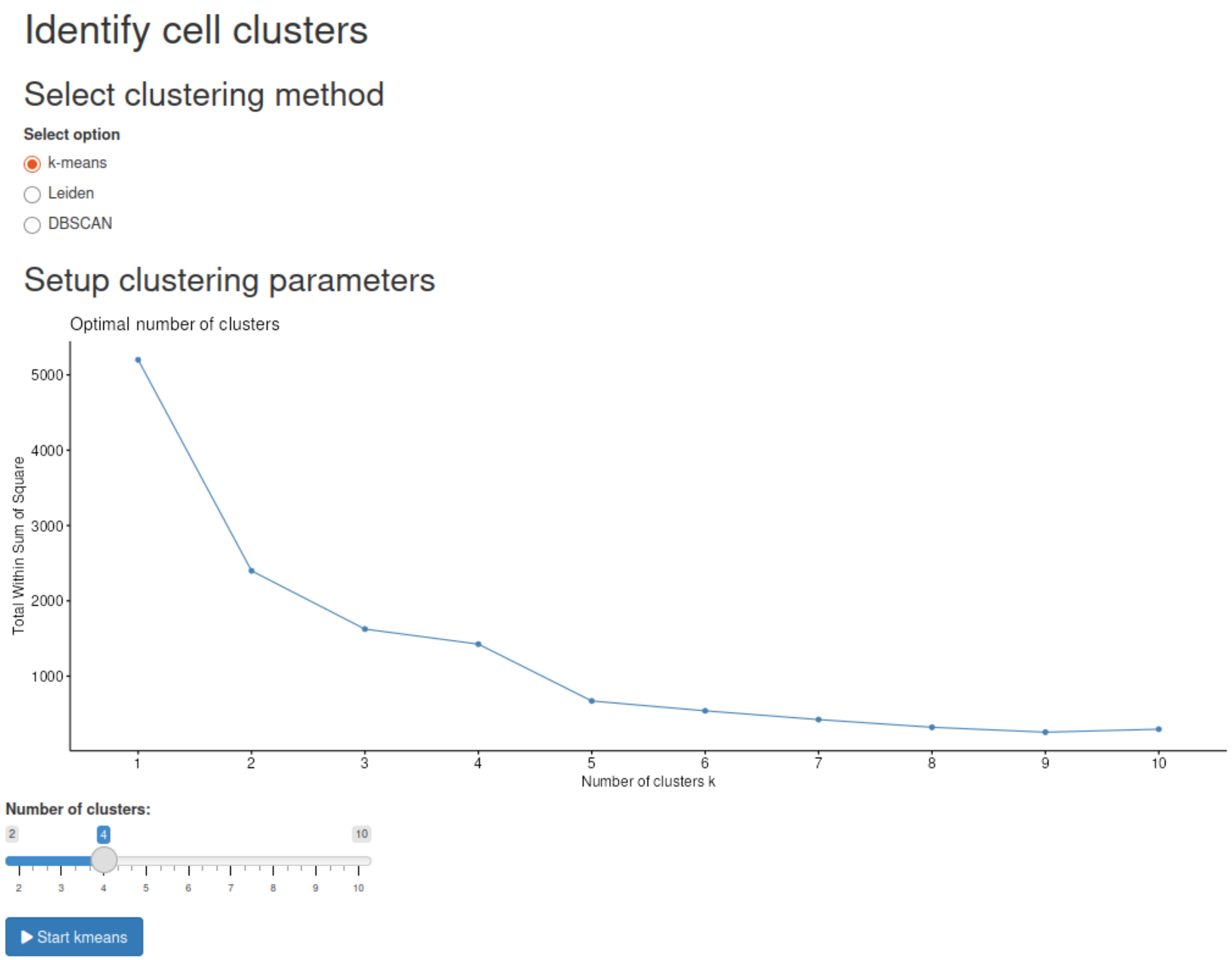

Figure S11: Interactive cell clustering. An elbow plot provides information on the optimum number of clusters for k-means clustering.

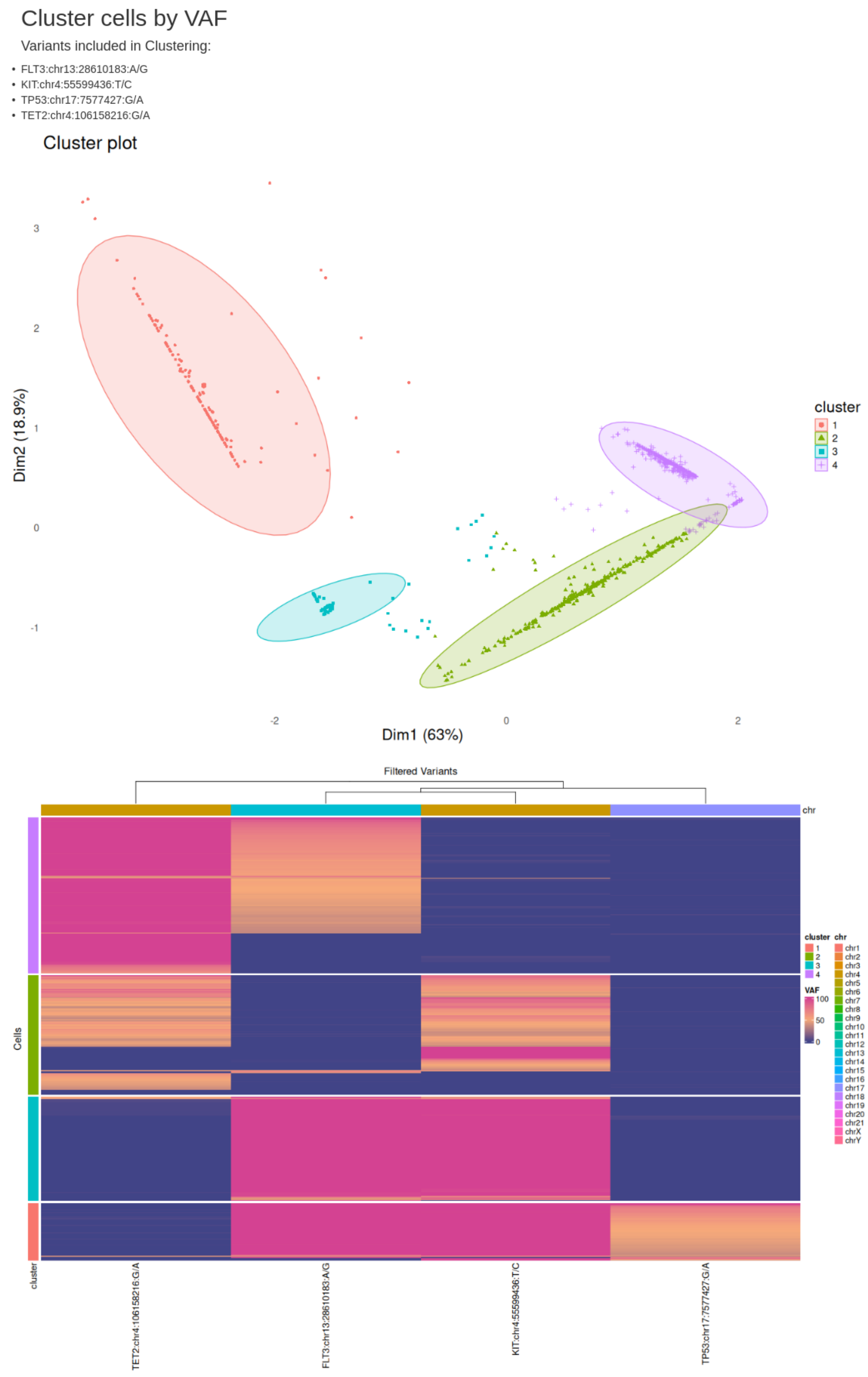

Figure S12: Cell clustering results. Cells are clustered by variant allele frequency, considering the set of user-selected variants (here: k-means clustering; principal component analysis). An updated heatmap, showing the variant allele frequency of the selected variants across filtered cells, is provided, now additionally annotated by cluster.

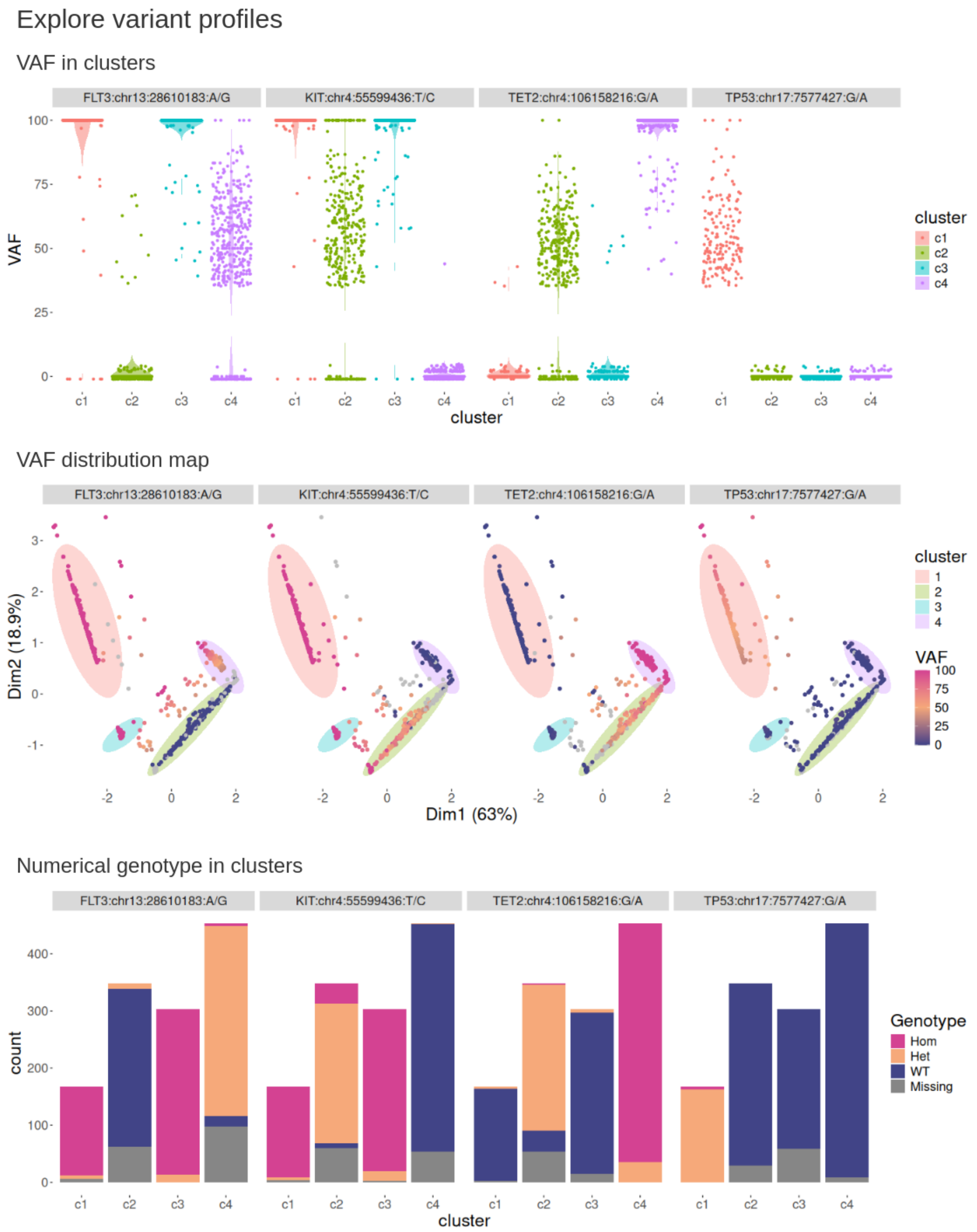

Figure S13: Variant profiles in cell clusters. For every selected variant, a set of three plots is depicted below each other. Scatter plots with underlying violin plots show the distribution of the variant allele frequency (VAF) per cluster. The VAF distribution is additionally displayed in principal component analysis plots, showing the identified clusters in the background. Deducing the genotypes from the observed VAFs per cell, their distribution per cluster is visualized as barplots.

2 Supplementary Tables

Table S1: Comparison of main features across different tools for scDNA-seq quality and variant analysis.

**Feature**	**scafari**	**optima[1]**	**mosaic[2]**	**scDNA[3]**
Programming language	R	R	Python	R
Availability	Bioconductor	GitHub	pip	GitHub
Graphical user interface	Yes	No	No	No
Basic quality statistics	Yes	Yes	Yes	Yes
Amplicon annotation	Yes	No	No	No
Variant filtering	Yes	Yes	Yes	Yes
Variant annotation	Yes	Yes	Yes	Yes

Table S2: Comparison of variants filtered by scafari, mosaic and optima using default filtration.

ID	Gene	Protein	Coding Impact	Function	DANN	ClinVar	dbSNP	gnomAD (%)	scafari	mosaic	optima
chr1-115256513-G-A	NRAS	NRAS:p.A66=	synonymous	coding	0.8080	-	rs2101741967		FALSE	TRUE	FALSE
chr1-115256669-G-A	NRAS	NRAS:p.p.?		intronic	0.4857	Benign	rs969273		TRUE	TRUE	TRUE
chr2-25458546-C-T	DNMT3A	DNMT3A:p.p.?		intronic	0.5510	Benign	rs2304429	51.38	TRUE	TRUE	TRUE
chr2-25470426-C-T	DNMT3A	DNMT3A:p.p.?		intronic	0.6352	-	rs142243425	0.07	TRUE	TRUE	TRUE
chr2-209113192-G-A	IDH1	IDH1:p.G105=	synonymous	coding	0.6809	Benign	rs11554137	5.06	TRUE	TRUE	TRUE
chr3-128200690-G-A	GATA2	GATA2:p.A372V	missense	coding	0.9994	-	-		FALSE	TRUE	FALSE
chr3-128200806-G-A	GATA2	GATA2:p.p.?		intronic	0.9152	Benign	rs11708606	15.64	TRUE	TRUE	TRUE
chr4-55599436-T-C	KIT	KIT:p.p.?		intronic	0.5625	Benign	rs1008658		TRUE	TRUE	TRUE
chr4-55602765-G-C	KIT	KIT:p.L862=	synonymous	coding	0.4692	-	rs3733542	10.78	TRUE	TRUE	TRUE
chr4-106154990-T-TATAG	TET2	TET2:p.p.?		intronic		-	rs58201766		FALSE	TRUE	FALSE
chr4-106156983-G-A	TET2	TET2:p.E628=	synonymous	coding	0.3203	Benign	rs35695427	0.41	TRUE	TRUE	TRUE
chr4-106158216-G-A	TET2	TET2:p.S1039=	synonymous	coding	0.5878	Benign	rs3796927	3.21	TRUE	TRUE	TRUE
chr4-106190862-T-C	TET2	TET2:p.H1380=	synonymous	coding	0.4049	Benign	rs3733609	3.41	TRUE	TRUE	TRUE
chr4-106194275-T-C	TET2	TET2:p.p.?		intronic	0.6730	-	-		FALSE	TRUE	FALSE
chr4-106196792-T-C	TET2	TET2:p.C1709R	missense	coding	0.9695	-	-		FALSE	TRUE	FALSE
chr4-106196829-T-G	TET2	TET2:p.L1721W	missense	coding	0.9315	Benign	rs34402524	11.56	TRUE	TRUE	TRUE
chr4-106197469-G-A	TET2	TET2:p.K1934=	synonymous	coding	0.4149	Likely Benign	rs569967455	0.003	TRUE	TRUE	TRUE
chr7-148504854-A-AGACTT	EZH2	EZH2:p.p.?		intronic		Benign	rs560966145		TRUE	TRUE	TRUE
chr7-148506064-A-G	EZH2	EZH2:p.p.?		intronic	0.7012	Benign	rs740949		TRUE	TRUE	TRUE
chr7-148508833-A-G	EZH2	EZH2:p.p.?		intronic	0.5855	Benign	rs2072407	66.89	TRUE	TRUE	TRUE
chr7-148515101-GC-G	EZH2	EZH2:p.R369Sfs*55	frameshift	NMD-coding		-	-		FALSE	TRUE	FALSE
chr7-148526923-A-G	EZH2	EZH2:p.V127=	synonymous	coding	0.7426	-	-		FALSE	TRUE	FALSE
chr7-148529659-A-C	EZH2	EZH2:p.p.?		intronic	0.8712	-	-		FALSE	TRUE	FALSE
chr7-148543525-A-G	EZH2	EZH2:p.p.?		intronic	0.6465	Benign	rs10274535	70.23	TRUE	TRUE	TRUE
chr11-32417945-T-C	WT1	WT1:p.R374=	synonymous	coding-splicing	0.5301	Benign	rs16754	24.34	TRUE	TRUE	TRUE
chr11-32421651-T-C	WT1	WT1:p.p.?		intronic	0.3269	-	rs111692843		TRUE	TRUE	TRUE
chr12-112888239-C-T	PTPN11	PTPN11:p.H85=	synonymous	coding	0.5426	Benign	rs61736914	0.58	TRUE	TRUE	TRUE
chr13-28597686-G-A	FLT3	FLT3:p.p.?		intronic	0.5591	-	rs76991010		TRUE	TRUE	TRUE
chr13-28610183-A-G	FLT3	FLT3:p.p.?		intronic-splicing	0.5447	Benign	rs2491231	70.97	TRUE	TRUE	TRUE
chr17-7572996-G-T	TP53	TP53:p.S371=	synonymous	coding	0.8645	-	rs765530090		FALSE	TRUE	FALSE
chr17-7577427-G-A	TP53	TP53:p.p.?		intronic	0.6903	Benign	rs12947788		TRUE	TRUE	TRUE
chr17-7577581-A-G	TP53	TP53:p.Y234H	missense	coding	0.9983	-	rs864622237		TRUE	TRUE	TRUE
chr17-7578115-T-C	TP53	TP53:p.p.?		intronic	0.6480	Benign	rs1625895	86.33	TRUE	TRUE	TRUE
chr17-7578176-C-T	TP53	TP53:p.p.?		intron-spl-ACMG	0.9945	-	rs863224499		TRUE	TRUE	TRUE
chr17-7578211-C-T	TP53	TP53:p.R213Q	missense	coding	0.9994	-	rs587778720	0.0004	TRUE	TRUE	TRUE
chr17-7578523-T-TG	TP53	TP53:p.Q136Pfs*13	frameshift	NMD-coding		-	-		TRUE	TRUE	TRUE
chr17-7580052-C-T	TP53	TP53:p.p.?		intronic	0.2569	Benign	rs8079544		TRUE	TRUE	TRUE
chr20-31021461-C-T	ASXL1	ASXL1:p.A487V	missense	coding	0.9959	-	rs2123263727		FALSE	TRUE	FALSE
chr20-31022288-C-A	ASXL1	ASXL1:p.Y591*	nonsense	coding	0.9967	Likely Pathogenic	rs371369583	0.0008	TRUE	TRUE	TRUE
chr21-44514571-G-A	U2AF1	U2AF1:p.p.?		intronic-splicing	0.6520	-	-		FALSE	TRUE	FALSE

References

[1] Dong P, Griffard R, Yellapu NK *et al.* optima: an open-source R package for the Tapestri platform for integrative single cell multiomics data analysis. *Bioinformatics*, 39(10):btad611, 2023.

[2] Mission Bio. *mosaic 3.7.0*, 2025. https://github.com/MissionBio/mosaic.

[3] Miles LA, Bowman RL, Merlinsky TR *et al.* Single-cell mutation analysis of clonal evolution in myeloid malignancies. *Nature*, 587(7834):477–482, 2020.
